# Intragenus F1-hybrids of African weakly electric fish (Mormyridae: *Campylomormyrus tamandua ♂ *× *C. compressirostris* ♀) are fertile

**DOI:** 10.1007/s00359-020-01425-7

**Published:** 2020-05-28

**Authors:** Yevheniia Korniienko, Linh Nguyen, Stephanie Baumgartner, Marianne Vater, Ralph Tiedemann, Frank Kirschbaum

**Affiliations:** 1 Faculty of Life Sciences, Albrecht Daniel Thaer-Institute of Agricultural and Horticultural Sciences, Unit of Biology and Ecology of Fishes, Humboldt University of Berlin, Philippstr. 13, Haus 16, 10115 Berlin, Germany; 2 Institute of Biochemistry and Biology, Unit of Evolutionary Biology/Systematic Zoology, University of Potsdam, Karl-Liebknecht-Str. 24-25, Haus 26, 14476 Potsdam, Germany; 3 Institute of Biochemistry and Biology, Unit of General Zoology, University of Potsdam, Karl-Liebknecht-Str. 24-25, Haus 26, 14476 Potsdam, Germany

**Keywords:** Mormyridae, *Campylomormyrus*, F1-hybrids, F2-hybrids, Fertility

## Abstract

Hybridization is widespread in fish and constitutes an important mechanism in fish speciation. There is, however, little knowledge about hybridization in mormyrids. F1-interspecies hybrids between *Campylomormyrus tamandua ♂ *× *C. compressirostris* ♀ were investigated concerning: (1) fertility; (2) survival of F2-fish and (3) new gene combinations in the F2-generation concerning the structure of the electric organ and features of the electric organ discharge. These F1-hybrids achieved sexual maturity at about 12–13.5 cm total length. A breeding group comprising six males and 13 females spawned 28 times naturally proving these F1-fish to be fertile. On average 228 eggs were spawned, the average fertilization rate was 47.8%. Eggs started to hatch 70–72 h after fertilization, average hatching rate was 95.6%. Average mortality rate during embryonic development amounted to 2.3%. Average malformation rate during the free embryonic stage was 27.7%. Exogenous feeding started on day 11. In total, we raised 353 normally developed larvae all of which died consecutively, the oldest specimen reaching an age of 5 months. During survival, the activities of the larval and adult electric organs were recorded and the structure of the adult electric organ was investigated histologically.

## Introduction

There is increasing evidence for hybridization constituting an important speciation mechanism both in plants and animals (Stebbins 1950; Dowling and Secor 1997; Barton 2008). Interspecies hybrids are characterized by new gene combinations leading to specific anatomical, physiological and behavioural features. While hybrids among closely related lineages may exhibit heterosis, i.e., high viability, fast growth, better physiological conditions and higher production (Darwin 1876; Wakchaure et al. 2015), genetic incompatibilities typically occur in hybrids of more distantly related lineages and may constitute a postzygotic isolation mechanism among species, leading to low viability, high mortality rate and low fertility or sterility (Tischler 1907; Walker 1926; Mayr 1970; Wu 1992).

Numerous examples of hybrids between species are known from birds (Grant and Grant 1992) and mammals (Gray 1972). The most well-known hybrids of mammals are the red wolf (hybridization of the gray wolf *C. lupus* and coyotes) (Wayne and Jenks 1991), grolar bear (cross between male braun bear and female polar bear) (Cahill et al. 2013; Kumar et al. 2017), liger (cross between male lion and female tiger) (Gray 1972; Li et al. 2016), leopon (cross between male leopard and female lion), zorse (cross between male horse and female zebra), zony (cross between male zebra and female horse), camas (cross between male dromedary camel and female llama) (Bonnicksen 2009), wolphin (cross between male false killer whale *Pseudorca crassidens* and female bottlenose dolphin *Tursiops truncatus*) (Klemm et al. 1995). However, most of these hybrids are uncommon in nature and have been bred for genetical and medical investigations (Bonnicksen 2009).

Among fish, natural hybridization occurs more commonly than in other vertebrate taxa (Schwartz 1972; Campton 1987; Leary et al. 1995; Allendorf and Waples 1996). In more than 19.7% of fish families worldwide hybrids have been observed (Nelson 1994). In marine species, hybrids are widely found in the families belonging to the Atherinomorpha and Percomorpha (Schwartz 1972, 2001). Hybrids in freshwater fish have been documented in 30 families and mainly occur in the Acipenseridae (sturgeons), Cyprinidae (minnows or carps), Esocidae (pikes), Salmonidae (salmonids), Centrarchidae (sunfishes) and Percidae (perches) (Schwartz 1981, 2001). In addition, hybrids are often found in families, the representatives of which inhabit both freshwater and marine habitats, such as the Cichlidae (cichlids), Poeciliidae (poeciliids) and Moronidae (temperate basses) (Schwartz 2001).

Molecular genetic studies have repeatedly shown that hybridisation in fish is a common phenomenon and is often detected as an ancient introgression (Schliewen and Klee 2004; Herder et al. 2006; Schwarzer et al. 2012a, b; Meier et al. 2017; MacGuigan and Near 2019).

In the freshwater family of the weakly electric Mormyridae (elephant fish) with more than 200 species (Lavoué et al. 2003), natural hybrids are only rarely observed. Natural hybridization has been found only in the genus *Paramormyrops,* which contains 22 species (Lavoué et al. 2008): hybrids were observed between morphs of the *Paramormyrops magnostipes* species complex characterized by differences of their electric organ discharge (EOD) (Arnegard et al. 2005). In *Paramormyrops kingsleyae* natural hybrids were observed among morphs occurring in geographic proximity (Gallant et al. 2011). Sullivan et al. (2004) discuss past introgression (i.e., hybridisation) in *Paramormyrops* inferred from mitochondrial data.

Recently, intra- and inter-genus-hybrids between species of the mormyrid genus *Campylomormyrus* and the genera *Gnathonemus*, *Mormyrus* and *Hyppopotamyrus* have been produced by artificial reproduction and investigated concerning the anatomy of the electric organs (EOs) and the EODs, relative to those of the parent species (Kirschbaum et al. 2016). For example, *C. compressirostris* and *C. tamandua* possess adult electric organs with considerable anatomical differences: the electrocytes of *C. compressirostris* have a posterior position of the main stalk, whereas those of *C. tamandua* are characterized by an anterior position of the stalks, which are smaller and penetrate the electrocytes. Both species produce short duration EODs of about 200 µs (*C. compressirostris*) and 300 µs (*C. tamandua*). The EOD of *C. compressirostris* is biphasic, while *C. tamandua* produces a triphasic EOD comprising a first short, head negative phase followed by a head positive and head negative phase (Paul et al. 2015). The F1-hybrids produce an EOD with four phases and they possess an electric organ with a posterior position of the main stalk and double penetrations (Kirschbaum et al. 2016).

The aims of this study were: (1) to find out if these F1-hybrids were fertile and were able to produce F2-fish; (2) to investigate the anatomy of the EO and the EOD in F2-fish which are expected to carry new gene combinations for these features; (3) to determine segregation of phenotypic traits (EO histology, EOD), pointing towards novel allelic combinations at underlying genetic loci.

## Materials and methods

### Animals and experimental tanks

**Fig. 1:**
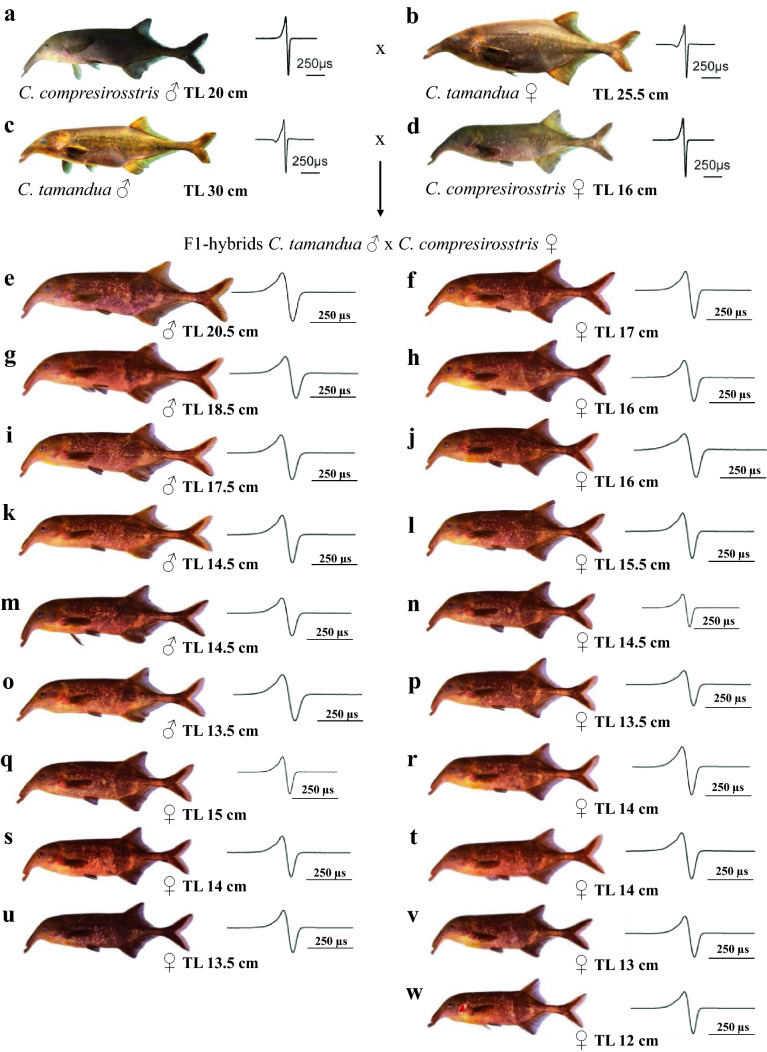
Morphology of *C. tamandua and C. compressirostris* and of their F1-hybrids. The appropriate electric organ discharges are also indicated. Note that the EODs of the parent species and those of the hybrids are not shown at the same time scale. Males can be differentiated from the females by the modified anal fin

The F1-hybrids between *C. tamandua ♂ *× *C. compressirostris* ♀, which we used in this study, are those fish which had been used before for the artificial reproduction of F2-hybrids (Kirschbaum et al. 2016). They comprised 32 fish. We devided them in two breeding groups of 19 (Fig. 1) and 13 fish, respectively.

The breeding tanks had the following dimensions: 155 × 70 × 60 cm. These tanks were equipped with a foam filter, a recurrent water pump behind the filter, an air supplier and plastic tubes of sizes 50 × 30 cm and of different diameter about 5–10 cm as hiding places. To protect eggs from foraging a grid was placed on the bottom of the aquarium.

Adult fish were fed twice daily with live *Chaoborus* (*Corethra*) and chironomid larvae or frozen larvae. To raise the fry, eggs were collected from inside the protecting cage the morning after spawning and were put into Petri dishes (*ø* = 8.6 cm, 1.5 cm high) with various densities between 5 and 10 eggs per dish. One day old eggs were checked if they were fertilized or unfertilized. Only fertilized eggs were selected for further incubation in water of the breeding tank. Incubation was performed in an incubator at 27 ± 0.5 °C or at room temperature (23–25 °C). After hatching, free embryos were transferred into new Petri dishes and tap water (conductivity ca. 700 µs/cm; pH ca. 8) was added gradually to the water of the breeding tank to rear them. One week after hatching the free embryos could be raised in tap water. About 1 month after hatching, the larvae were transferred into quadrangular petri dishes (10 × 10 × 2 cm) and raised at room temperature. From the beginning of exogenous feeding up to about 30–40 mm long juveniles, newly hatched *Artemia nauplii* were offered as food. Thereafter, small chironomid larvae were additionally provided. Larvae and juvenile fish between 15 and 40 mm total length were kept in plastic boxes (20 × 10 × 5 cm) equipped with aeration. Thereafter, the fish were transferred into small aquaria (30 × 20 × 20 cm) equipped with a foam filter, aeration and various types of hiding-places: broken plant pots, PVC pipes of 3 cm diameter, gravel (ca. 2–4 cm) and drift wood.

### Manipulation of environmental factors

For breeding, the fish were subjected to a decrease of water conductivity over several weeks to imitate the rainy season conditions (Schugardt and Kirschbaum 2004). The temperature ranged between 25 and 28 °C.

Temperature and water conductivity were measured daily with a conductivity meter (WTW Multi 340i; WTW Wissenschaftlich–Technische Werkstätten, 82,362 Weilheim–Germany). pH was measured once a week with a pH meter (WTW Multi 340i; with the PH electrode SenTix 41; WTW Wissenschaftlich–Technische Werkstätten, 82,362 Weilheim–Germany).

Photoperiod was held constant at 12L:12D (light from 6:00 a.m. to 6:00 p.m.) by a time switch. In summer, however, some diffuse light penetrated the opaque windows of the aquarium room.

### Histological techniques

The EOs were subjected to anatomical examinations via light microscopy. Three F2-hybrids were fixed shortly after death by immersion in 4% formalin and the caudal peduncle was cut off. Dehydration and embedding in paraffin followed conventional techniques (Mulisch and Welsch 2010). The EO was sectioned serially at 4–6 µm thickness in the transverse or sagittal plane using a Leica 2035 Biocut microtome. Sections were mounted on glycerin/albumin-coated slides. Following deparaffination and rehydration, sections were stained with Azan or HE (Mulisch and Welsch 2010), dehydrated and coverslipped with DePeX mounting medium (Sigma-Aldrich). Sections were investigated with a Leica microscope DM4000B. Pictures were taken and processed using the Leica DFC 480 camera together with the Leica IM software version 4.0.

### Photography

Eggs, embryos, free embryos and up to 15 mm long larvae were recorded with a Leica S6E binocular fitted with a Canon Powershot S50 digital camera. Photos of older larvae, juveniles and adults were taken with a digital camera Canon EOS 350D and Canon EOS 100D. Pictures of eggs, embryos and early free embryos were taken from live specimens. All photos of later free embryos, larvae, juveniles, and adults were documented from anaesthetized fish.

### Oscilloscope recordings

The development of the EOD of the individual specimens was displayed and recorded using a Tektronix TDS 3012B digital phosphor oscilloscope (maximum sampling rate 1.25 GS/s; 9 bit vertical resolution) with a Tektronix ADA 400A differential preamplifier (variable gain from 0.1 × up to 100×; bandwidth 100 Hz – > 1 MHz). We used the > 1 MHz position to avoid any limitation during recording. The oscilloscope and preamplifier were AC coupled.

Larvae up to a size of ca. 30 mm were placed into a small cube-like plastic container of 7.5 cm × 5 cm with water level of 1.5 cm; bigger larvae, juvenile and adult fish were measured in a bigger plastic container of 30 cm × 15 cm with water level from 3 to 10 cm. Containers for EOD measurements were filled with water from the keeping tank (conductivity 600–800 µs/cm, temperature 23–25 °C, pH 6–8) and equipped with a compartment adjustable to restrict the movement of the fish. For EOD measurement, the positive electrode was positioned a few centimeter away from the head of the fish and the negative electrode beyond the caudal fin.

EODs were recorded twice a month during the larval stage and once a month during the juvenile stage.

## Results

### Breeding experiments with F1-hybrids (*C. tamandua ♂ *× *C. compressirostris ♀*)

**Fig. 2:**
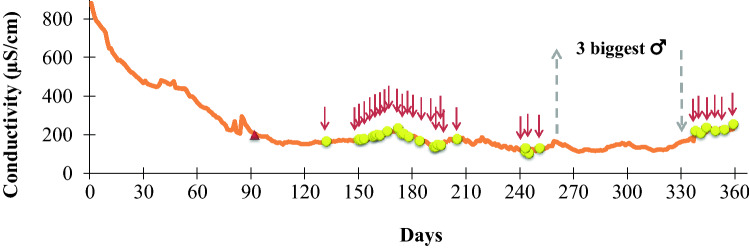
Breeding experiment with 19 F1-hybrids (6 males, 13 females) (*C. tamandua ♂* × *C. compressirostris* ♀) for a period of 360 days. Red arrows indicate natural spawnings. Oviposited eggs were always fertilized (see Table 1). Triangle indicates successful artificial reproduction (see Kirschbaum et al. 2016). On day 260 the three biggest males were taken out of the breeding tank and were returned on day 337 (grey arrows). Note the stop and start of spawning activity

The 32 F1-hybrids were divided into two potential breeding groups with 13 and 19 fish, respectively. With the breeding group comprising 19 fish (Fig. 1), we conducted a breeding experiment by lowering the water conductivity for a period of 360 days (Fig. 2; Table 1). During this period 28 natural spawnings occurred. The number of spawned eggs ranged between 16 and 1039 (average 228), the fertilization rate between 9.0 and 87.4% (average 47.8%). The weakly sticky eggs (diameter 1.2–1.3 mm) started to hatch 70–72 h after fertilization, the range of hatching rate was between 73.7 and 100% (average 95.6%).

**Table 1:** Some features of early developmental aspects of F2-hybrids following 28 spawnings (see Fig. 2) of a group of 19 F1-hybrids (*C. tamandua ♂* × *C. compressirostris* ♀) (6 males, 13 females) over a period of 229 days

No of spawn-ing	Date of spawning	Day of experiment	Conduct. (μs/cm)	Interval towards the preceding spawning (days)	No of collected eggs	No of fertilized eggs	Fertilization rate (%)
1	23.01.17	133	166.1	–	31	9	29.0
2	10.02.17	151	176.0	18	168	100	59.5
3	11.02.17	152	175.7	1	762	143	18.8
4	13.02.17	154	178.4	2	440	122	27.7
5	18.02.17	159	191.3	5	1039	93	9.0
6	20.02.17	161	199.9	2	114	75	65.8
7	22.02.17	163	197.4	2	163	90	55.2
8	26.02.17	167	218.0	3	36	19	52.8
9	04.03.17	173	234.0	5	83	30	36.1
10	06.03.17	175	207.0	2	379	128	33.8
11	07.03.17	176	206.0	1	497	275	55.3
12	09.03.17	178	189.0	2	30	12	40.0
13	16.03.17	185	167.9	7	337	141	41.8
14	24.03.17	193	138.5	1	127	65	51.2
15	25.03.17	194	136.8	1	175	8	4.6
16	26.03.17	195	149.0	1	666	289	43.4
17	28.03.17	197	145.7	2	95	55	57.9
18	06.04.17	206	178.3	9	16	8	50.0
19	14.05.17	244	114.5	38	20	15	75.0
20	16.05.17	246	103.3	2	83	58	69.9
21	22.05.17	252	130.7	6	192	58	30.2
22	17.08.17	339	220.0	87	154	78	50.6
23	20.08.17	342	208.0	3	130	98	75.4
24	23.08.17	345	237.0	3	183	160	87.4
25	28.08.17	350	220.0	5	33	15	45.5
26	02.09.17	355	227.0	5	55	30	54.5
27	07.09.17	360	255.0	5	103	36	34.9
28	09.09.17	362	248.0	2	280	233	83.2
Average				3	228	87	47.8

For the calculation of the average concerning the spawning intervals the three very high values were not integrated

The 19 specimens of this breeding group comprised 6 males and 13 females (Fig. 1). As mormyrids spawn during the night (Kirschbaum 1987; Schugardt and Kirschbaum 1998), we were not able to observe spawning behavior in these fish.

On day 260 of the breeding experiment (Fig. 2) we took out the three largest males (Fig. 1) for subsequent backcross experiments (not reported in this paper). From that day on no further spawning activity was observed. On day 337 of the breeding experiment we returned the three large males to the original breeding group and a few days later spawning started again (Fig. 2).

From the 28 natural spawnings obtained from the breeding group with the 19 fish, we selected 11 spawnings to determine developmental parameters (Table 2).

**Table 2:** Comparative data of F2-hybrids concerning fertilization rate, mortality during embryonic development, hatching rate and proportion of malformation during the free embryonic stage of one artificial reproduction and 11 natural spawnings of the breeding group of 19 F1-hybrids (*C. tamandua ♂* x *C. compressirostris* ♀) (see Fig. 2 and Table 1)

Date of artificial reprod. resp. natural spawning (nat. sp.)	Kind of reprod	No of eggs	No of fertil. eggs (%)	No of dead embryos during embryo- nic stage (%)	No of hatched embryos (%)	No of free embryos with malforma- tions (%)	No of normal larvae at the beginning of exogenous feeding (raising experiments)
*14.12.2016*	*Artificial*	*195*	*40 (32.7)*	*0 (0)*	*39 (97.5)*	*10 (25.6)*	*29*
*23.01.2017*	*nat. sp.*	*31*	*9 (71)*	*0 (0)*	*9 (100)*	*1 (11.1)*	*8*
*10.02.2017*	*nat. sp.*	*168*	*100 (59.5)*	*6 (6)*	*91 (96.8)*	*31 (34.1)*	*60*
*11.02.2017*	*nat. sp.*	*762*	*143 (18.8)*	*1 (0.7)*	*141 (99.3)*	*16 (11.3)*	*125*
*13.02.2017*	*nat. sp.*	*440*	*122 (27.8)*	*9 (7.4)*	*112 (99.1)*	*34 (30.1)*	*78*
*26.02.2017*	*nat. sp.*	*36*	*19 (52.5)*	*0 (0)*	*14 (73.7)*	*6 (42.9)*	*8*
*22.05.2017*	*nat. sp.*	*192*	*58 (30.2)*	*1 (1.7)*	*55 (96.5)*	*10 (18.2)*	*45*
17.08.2017	nat. sp.	154	78 (50.6)	3 (3.8)	71 (94.7)	26 (37.3)	–
28.08.2017	nat. sp.	33	15 (45.5)	0 (0)	15 (100)	5 (33.3)	–
02.09.2017	nat. sp.	55	30 (54.5)	0 (0)	29 (96.7)	9 (31.6)	–
07.09.2017	nat. sp.	103	36 (34.9)	2 (5.6)	32 (94.1)	6 (18.8)	–
09.09.2017	nat. sp.	280	233 (83.2)	5 (2.1)	224 (98.2)	86 (38.4)	–
Average	*–*	204	74 (46.8)	2 (2.3)	69 (95.6)	20 (27.7)	353 (total no)

Italic background indicates those spawnings of which the larvae were raised

### Detailed ontogenetic description of the F2-hybrids

**Fig. 3:**
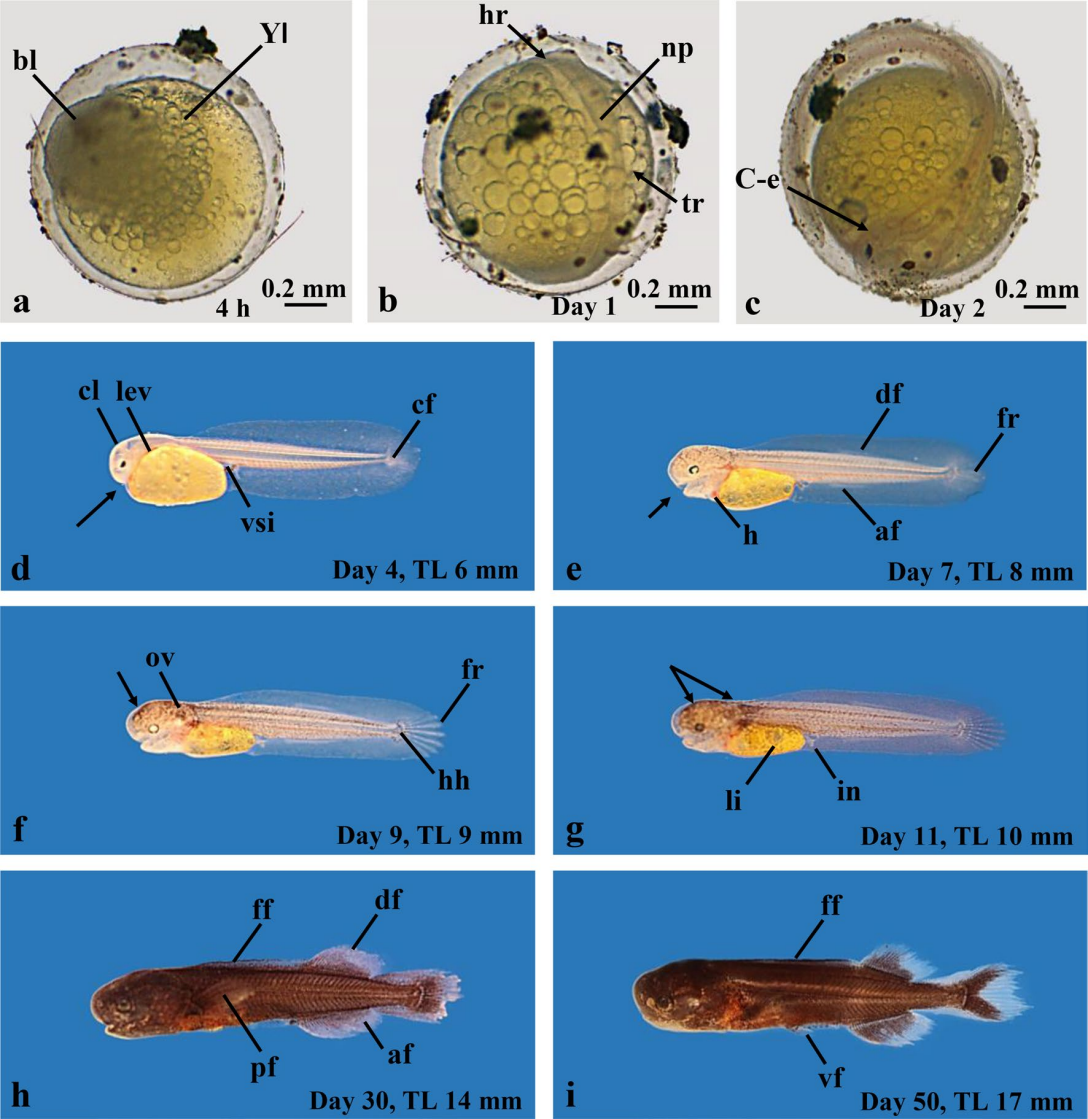
Early ontogenetic development of F2-hybrids (*C. tamandua ♂* × *C. compressirostris* ♀). **a** Three hours old egg with a blastula (bl). **b** One day old early embryo. **c** Two day old embryo prior to hatching. **d** Four day old free embryo just after hatching. **e** Seven day old embryo. The yolk sac has decreased in size, the eye is more developed than in the 7 day old embryo, the mouth (arrow) has opened, and dorsal (df) and anal fins (af) start to differentiate. **f** Nine day old embryo. Black pigment has started to develop on the head (arrow); fin rays of the caudal fin (fr) are recognisable. **g** 11 day old embryo at the start of exogenous feeding. Yolk sac is nearly entirely resorbed; head and eyes are well pigmented; liver (li) and intestine (in) are recognisable. **h** Thirty day old larva. Paired and unpaired fins are well developed; the body is fully pigmented. **i** Larva 50 days old; remnants of the embryological fin fold (ff) are still present on the caudal peduncle. *YI* layer of yolk syncytium, *np* neural plate, *hr* prospective brain region, *tr* prospective trunc region, *C-e* c-shaped embryo, *pf* pectoral fin, *vf* ventral fin, *ov* otic vesicle, *hh* hypural plates

In Fig. 3 we describe the basic morphological development of the F2-hybrids. One day old eggs show a dense mass of cells (a blastula; Fig. 3a); a day later the early embryo is visible (Fig. 3b), which at day 2 has progressed in development (Fig. 3c). The embryos hatch on day 3 (Fig. 3d) and are characterized by a large yolk sac, a large embryological fin fold and a small eye. Figure 3e shows the free embryo at day 7; the yolk sac has decreased in size, the eye is more developed, the mouth has opened and dorsal and ventral fins start to differentiate. In the 9 days old free embryo (Fig. 3f) black pigment has developed on the head, a gas vesicle is seen in the otic vesicle, the fin rays of the caudal fin are obvious and some skeletal elements of the caudal plate are visible. The 11 days old embryo (Fig. 3g) starts exogenous feeding, this stage is then termed larva. At day 30 (Fig. 3h) the larva is almost completely black and the paired and unpaired fins are well developed. This morphological development has progressed in the 50 days old larva (Fig. 3i). As there are still some remnants of the embryological fin fold present on the caudal peduncle this stage is still termed a larva. Further morphological development is shown in Fig. 7 along with the description of the EOD ontogeny.

**Fig. 4:**
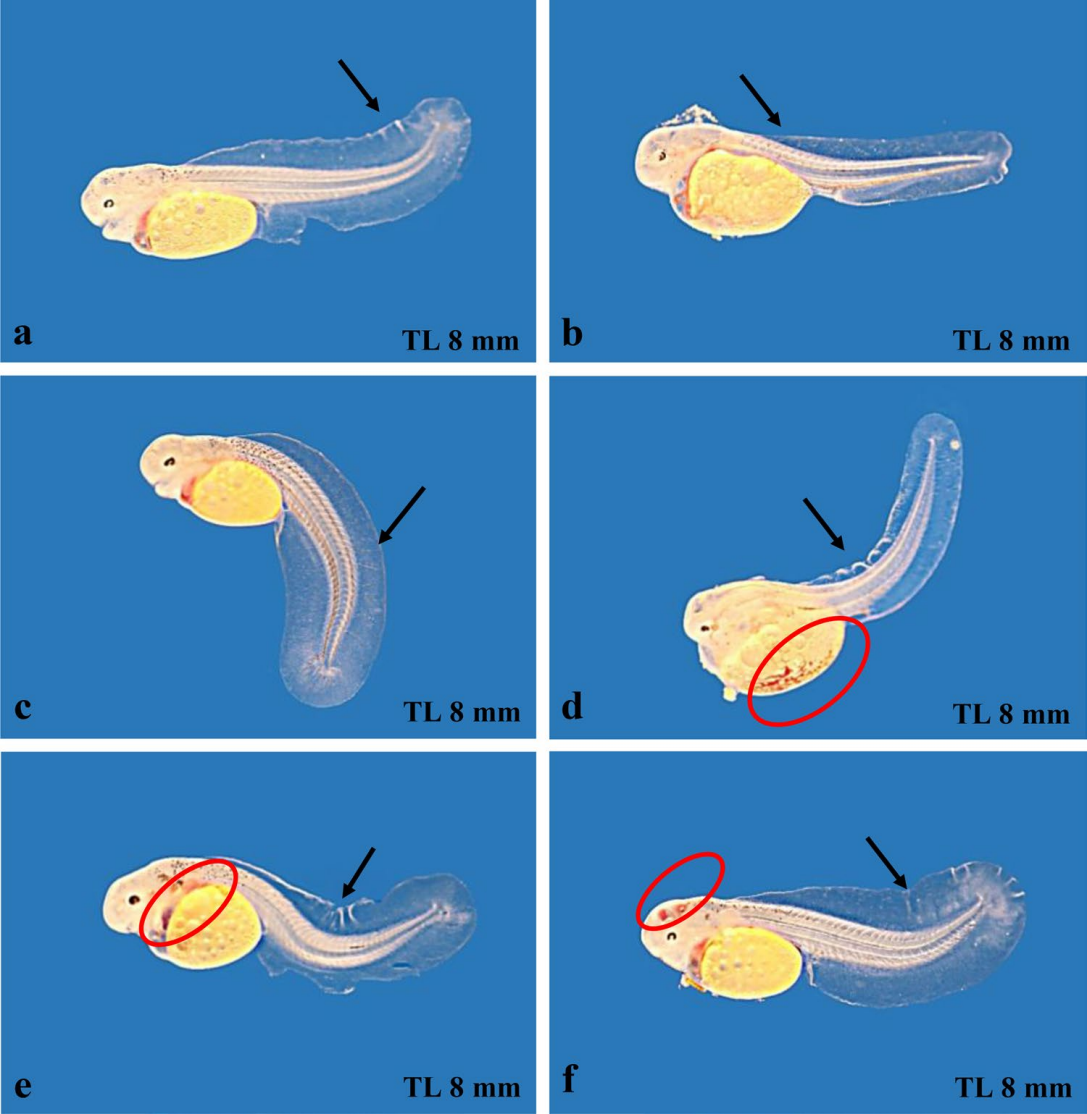
Malformations of free embryos of F2-hybrids (*Campylomormyrus compressirostris* female × *C. tamandua* male). Arrows point to malformations of the vertebral column; red circles indicate abnormalities of the circulatory system

In addition to the normally developing embryos and larvae we also observed some mortality during embryogenesis and the free embryonic stage (Table 2): mortality during the embryonic development ranged between 0 and 6% (mean 2.3%), and the proportion of malformation during the free embryonic stage ranged between 11.1 and 42.9% (mean 27.7%). In Fig. 4 we show some typical malformations during the free embryonic stage. The vertebral column is directed upwards (Fig. 4a), there may be a kick in the middle of the vertebral column (Fig. 4b), or the rear part of the body is turned downwards (Fig. 4c) or upwards (Fig. 4d). Some specimens are completely distorted and abnormalities in the blood circulation seem to occur (Fig. 4e, f). About 65% of the free embryos with malformations showed abnormalities concerning the vertebral column as shown in Fig. 4, about 20% are characterized by abnormalities of the circulatory system and ca. 15% had a malformed yolk sac. Specimens with these malformations in general did not survive for more than 14 days.

**Fig. 5:**
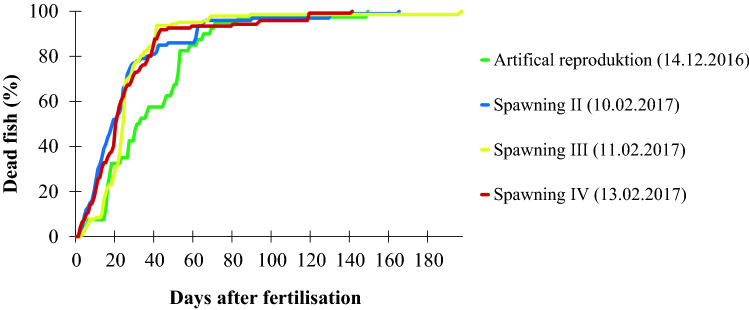
Cumulative mortality of four groups of F2-hybrids (*C. tamandua ♂* × *C. compressirostris* ♀). Group 1 (artificial reproduction; see Kirschbaum et al. 2016) consisted in the beginning of 40 fertilized eggs, group 2 of 100, group 3 of 143 and group 4 of 122 fertilized eggs. Note that most of the fish had died after about 60 days

For the determination of the cumulative mortality, we selected the fish from the artificial reproduction (40 fertilized eggs) (Kirschbaum et al. 2016) and three additional spawnings (100, 143, and 122 fertilized eggs, respectively) (Fig. 5; Table 1). The mortality during embryonic development is shown in Table 2. The four curves describing the cumulative mortality from the beginning of the fertilized stage on are quite similar and show that most of the fish die during the first 60 days of development. At that time, only about 10% of the hybrids had survived. Thereafter, only single individuals survived.

**Fig. 6:**
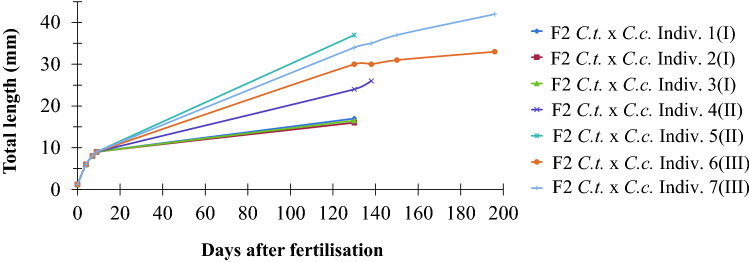
Growth curve of seven F2-hybrids (*C. tamandua ♂* × *C. compressirostris* ♀) from day 1 on up to their death

We selected seven individual F2-hybrids and monitored their individual growth (Fig. 6). Three fish from the artificial reproduction survived at maximum for about 50 days, two fish from the natural spawning from date 10.02.2017 (Table 2) survived for about 125 and 145 days, and two fish from the natural spawning from date 11.02.2017 survived for nearly 200 days at total lengths of 33 and 42 mm, respectively. The oldest specimen reached an age of about 190 days. This fish (No. 6) was measured several times between days 125 and days 190 and its growth curve indicates that growth slowed down during this period. This fish died at 42 mm total length from fungus infection after transfer into a small aquarium. During development the fish died for no obvious reasons, they in general seemed to be very sensitive to stress (e.g., during transfer from one jar to another).

### Morphological and EO development of individual fish

**Fig. 7:**
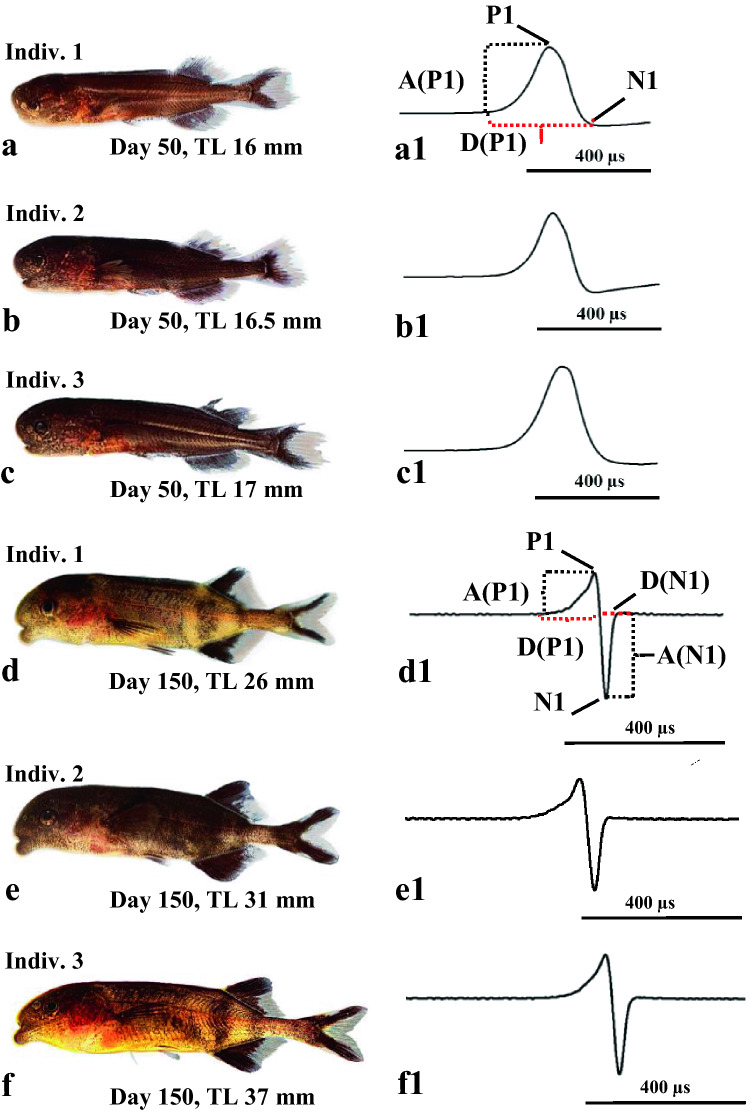
Ontogeny of morphology and electric organ discharge of three F2-hybrids (*C. tamandua ♂* × *C. compressirostris* ♀) shown at two developmental stages (50 and 150 days old)

One important aim of this study was the investigation of the anatomy of the EO and the ontogeny of the EOD of the F2-hybrids. As these hybrids were very sensitive and died prematurely, we could only monitor three individual fish at two different dates, at an age of 50 and 150 days, respectively (Fig. 7). When recorded at an age of 50 days, all three larvae still showed the larval EOD. However, the shape of the EOD differed slightly as did the duration of the head positive phase, amounting to about 300 µs (Table 3). The three fish, when measured 100 days later, had reached the juvenile stage, and all produced a biphasic EOD of about 170 µs duration, which is termed the juvenile EOD (Kirschbaum et al. 2016; Nguyen et al. 2017). Table 3 shows that the juvenile EODs of the three hybrids are quite similar concerning the total duration, the duration of the head positive and head negative phases and the ratio of the amplitude of the head positive and head negative phase. As these fish did not survive for a longer time, we could not continue to follow their EOD development.

**Table 3:** Electric organ discharge (EOD) features of the larval and adult EO of three F2-hybrids (*C. tamandua ♂* × *C. compressirostris* ♀) (shown in Fig. 7) concerning absolute amplitude (A) values, duration (D) of phases: the head-positive phase (P1) and the head-negative phase (N1) and relative amplitude ratios (R)

F2-hybrid specimen	Age (days)	Size (TL) (mm)	A (P1) (mV)	A (N1) (mV)	R: (P1/N1)	D (P1) (μs)	D (N1) (μs)	TD (μs)
Indiv. 1	50	16.0	334	–	–	320	–	–
Indiv. 2	50	16.5	316	–	–	283	–	–
Indiv. 3	50	17.0	416	–	–	339	–	–
Indiv. 1	150	26.0	10.3	20.8	0.5	119	54	173
Indiv. 2	150	31.0	6.6	11.8	0.56	123	43	166
Indiv. 3	150	37.0	8.3	14.4	0.58	118	55	173

The different phases of larval and adult EOD are shown in Fig. 7

**Fig. 8:**
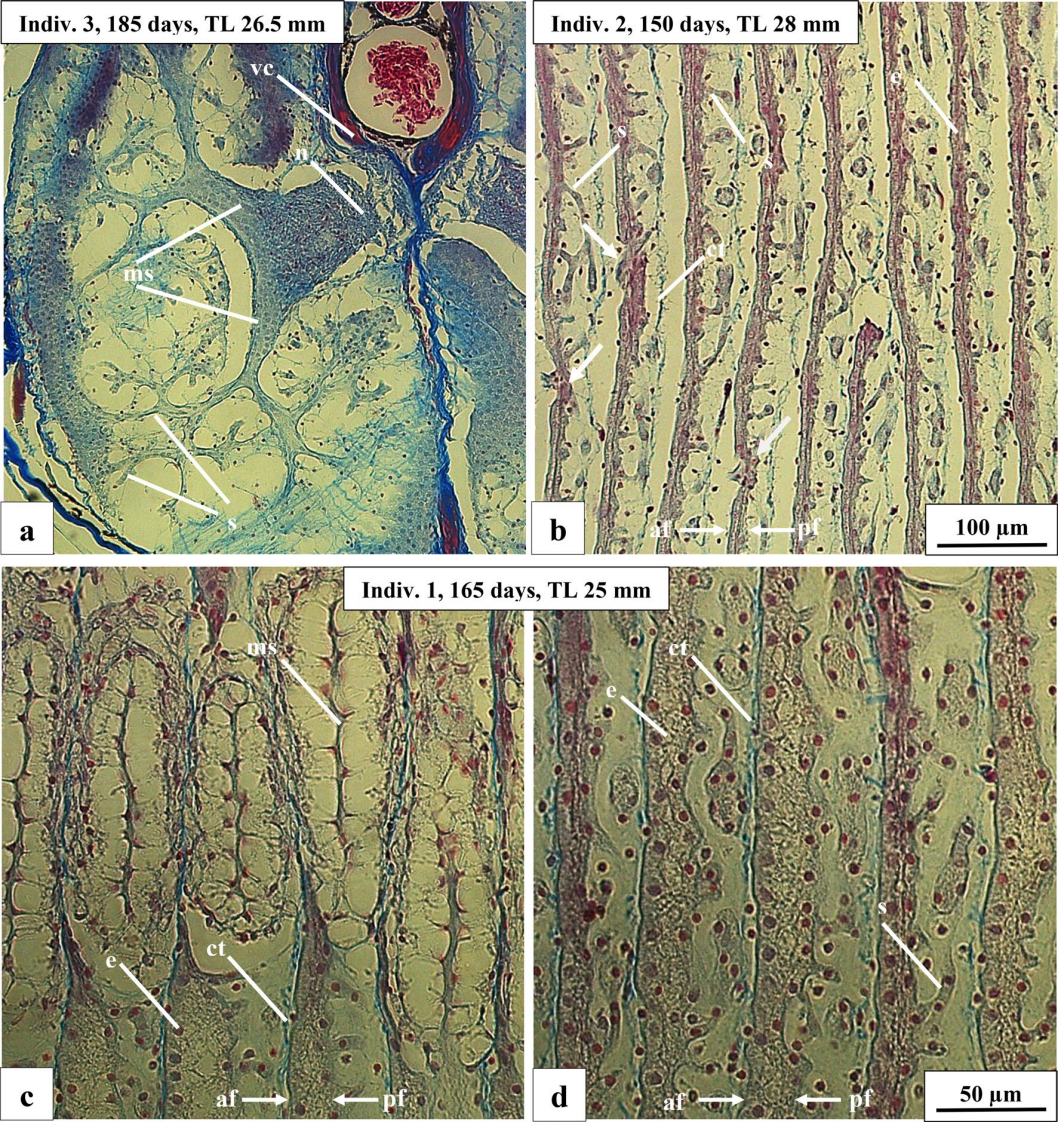
Photomicrographs of a cross section of a 26.5 mm long F2-hybrid (*C. tamandua ♂* × *C. compressirostris* ♀) and sagittal sections of two F2-hybrids, 28 and 25 mm long, respectively. **a** Cross section at higher magnification visualizing the extremely branched stalk system. **b** Regularly arranged electrocytes (e) showing stalks (s) and penetrating stalks (*arrows*). **c** Main stalk (ms) situated on the posterior face (pf) of the electroccyte. **d** Stalks originating at the posterior face (pf) of the electrocyte; penetrations are missing. *n* nerve, *vc* vertebral column

Because of the limited number of larger F2-hybrids, we did not sacrifice live fish for the histological examination of the adult EO, we instead fixed three fish shortly after their death. Figure 8a shows a cross section of the EO of a 26.5 mm long specimen. The branching network of the stalk system is very well visible. However, the cross section does not allow to observe the position of the stalks (anterior or posterior) nor do they reveal penetrations of the stalk.

We were able to investigate the EO in sagittal sections in two specimens (150 and 165 days old, respectively). The EO in both specimens shows a very regular arrangement of the individual electrocytes and the stalks originate at the posterior face of the electrocyte (Fig. 8b–d). In the 150 days old specimen (Fig. 8b) we found penetrations of the stalks through the main body of the electrocyte which we were not able to identify in the 165 day old specimen (Fig. 8c, d).

## Discussion

Hybridization constitutes an important mechanism in fish speciation. However, there is little knowledge about hybridization in mormyrids. We investigated F1-interspecies hybrids between *C. tamandua ♂ *× *C. compressirostris ♀* concerning: (1) fertility; (2) survival of F2-fish and (3) new allelic combinations in the F2-generation regarding the structure of the EO and features of the EOD.

These F1-hybrids proved to be fertile and spawned naturally over a prolonged period of time. The resulting F2-fish showed a very high mortality during the whole ontogeny and finally all fish had died after 5 months. During survival the activities of the larval and adult electric organs of a few specimens were recorded and the structure of the adult electric organ was investigated histologically.

### Fertility of F1-hybrids

The breeding experiment with the 19 F1-hybrids (Fig. 2) showed that these fish spawned very regularly over a prolonged period of time. Each spawning event led to fertilized eggs (Table 1). The average fertilization rate of 47.8% represents a high value among *Campylomormyrus* species (Nguyen et al. 2017). This shows that at least those specimens, which participated in the spawnings, were fertile. Probably, only the largest F1-hybrid male(s) were involved in the spawnings if we consider the spawning behavior of the hybrids to be comparable to the parent species *C. compressirostris* (Nguyen et al. 2017) and *C. tamandua* (Schugardt and Kirschbaum 1998). Indeed, when the three large males (Fig. 2) were taken out of the breeding tank, spawnings immediately stopped (Fig. 2), but started again when these males were put back to the breeding tank. We do not know why the three smaller males (Fig. 1) were not able to initiate successful spawnings. Possibly, they were not fertile. A definite answer about the fertility status of all the 19 specimens could only be gained via examination of the gonadal status of these specimens or breeding experiments with just a few specimens. However, breeding experiments with just a few specimens proved to be unsuccessful in *C. compressirostris*, because individual pairs did not successfully spawn (Nguyen et al. 2017).

Fertility of interspecific hybrids is quite common in fish and has been determined in 130 natural and 150 artificial performed crosses (Argue and Dunham 1999). Interspecific hybrids among salmonid fish such as *Oncorhynchus mykiss *× *Oncorhynchus clarki*, *Salvelinus fontinalis *× *Salvelinus namaycush* and *S. fontinalis *× *Salvelinus alpinus* are often fertile (Johnson et al. 1987). In the family Cichlidae hybrids of crosses of *Haplochromis. elegans *× *H. nubilus*, *H. “black lividus” *× *H. nubilus* and backcrosses F1 (*H. burtoni *× *H. nubilus*) × *H. burtoni,* F1 (*H. burtoni *× *H. nubilus*) × *H. nubilus* are also fertile (Crapon de Caprona and Fritzsch 1983). In the hybrid complex *Rutilus alburnoides* (Cyprinidae) research based on cytometric analysis of blood and sperm showed that both diploid and tetraploid hybrid males produced fertile sperm, but there are no data about fertility of triploid hybrids (Alves et al. 1999). In addition, F1-hybrids of roach × bream and backcrosses F1-hybrid × roach, roach × F1-hybrid and F1-hybrid × F1-hybrid were also shown to be fertile (Wood and Jordan 1987).

### Features of reproductive biology of hybrids and parent species

The F1-hybrids produced eggs of about 1.2 mm in diameter. In contrast, the parent species produce larger eggs: the eggs of *C. tamandua* have a diameter of 2 mm (Schugardt und Kirschbaum 1998; Kirschbaum und Schugardt 2006); those of *C. compressirostris* 2.3 mm (Nguyen et al. 2017). Stickiness of eggs is not easy to quantify. Still, the eggs of the F1-hybrids seemed to be less sticky than those of both parent species (Kirschbaum and Schugardt 2002; Nguyen et al. 2017).

The number of spawned eggs varies within and among the parent species: *C. compressirostris* spawned between 38 and 246 eggs per fractional spawning (Nguyen et al. 2017), whereas *C. tamandua*, depending on the size of the females, spawned between 383 (mean) and 1108 eggs (mean) (Schugardt und Kirschbaum 1998). The F1-hybrids spawned between 16 and 1039 eggs per fractional spawning (Table 1). During the artificial reproduction of the F1-hybrids the smaller female released 80 eggs and the larger one 115 eggs. We, therefore, conclude that more than one female must have participated in the spawning events (Table 1) when more than 115 eggs were deposited in our breeding groups. This occurred repeatedly during the breeding experiment (Table 1).

The minimum size for sexual maturity for the males (identification of the males was based on the presence of the male-typical anal fin anatomy, see Fig. 1) was in the range of 12–13.5 cm total length. This is comparable to the situation in *C. compressirostris* (Paul et al. 2015; Nguyen et al. 2017). The males grew larger than the females (Fig. 1). This is also typical for *Campylomormyrus* species (Nguyen et al. 2017). Our conclusion, therefore, is that all the female-type fish over 12 cm total length are females. This was indeed confirmed by the artificial reproduction experiment: both, the 12 and 13 cm female-type fish, released eggs (Kirschbaum et al. 2016).

### Mortality

Figure 5 and Table 2 show that the F2-hybrids had a high mortality. We suppose that three different kinds of deficiencies cause these deaths. (1) Mortality during embryogenesis was apparently caused by deficiencies during organogenesis (Table 2). (2) Many of the free embryos showed abnormalities of the vertebral column, the yolk sac, and the blood system (Fig. 4), which finally led to death. (3) Those fish which had started exogenous feeding around day 11 looked morphologically quite normal (Fig. 3g); however, most of them died up to an age of about 60 days (Fig. 5). They often looked a bit thin (Fig. 3h) and did not grow very well (Fig. 6). We suppose that in particular deficiencies at the level of feeding, nutrition and differentiation and function of the digestive tract are the main causes of death in addition to deficiencies at the level of the immune system (some fish died through infection with fungus).

We exclude raising conditions, in particular physico-chemical characteristics of the water, as causes for the death of the fish. We raised our larvae a few days after exogenous feeding in Berlin tap water at pH values around 8, conductivity values of about 700 µs/cm and temperatures between 23 and 28 °C. Under the same conditions several *Campylomormyrus* species including *C. compressirostris* were successfully raised (Nguyen et al. 2017) and about 80–95% of the larvae survived. Additional attempts to raise *C. compressirostris* and *C. tamandua* in Berlin tap water under semi-natural conditions revealed that at minimum 50% of the larvae survived (Hernandez Valencia 2019). About 20 ornamental freshwater fish species were raised successfully repeatedly in Berlin tap water (unpublished results).

### EOD and EO characteristics of the F2-hybrids

The main aim of this study was the attempt to obtain F2-hybrids and to follow the ontogeny of the discharge of the adult electric organ in different specimens, because in the F2-generation, new allelic combinations occur, potentially translating into differences in the EO and the EOD.

Figure 5 shows that most larvae had died about 60 days after fertilization. The three 50 day old larvae investigated (Fig. 7) produced a larval EOD (Fig. 7a1–c1). The shape of the EOD varies as does the duration of the head positive phase (Table 3). These individual differences in the shape and duration of the larval EOD are peculiar as the larval EOD in general, as seen in the different species, is quite stable (Westby and Kirschbaum 1977; Baier et al. 2006; Werneyer and Kramer 2006).

The adult EOD emerges in *Campylomormyrus* larvae at a much later stage than 50 days after hatching (Nguyen et al. 2017). Figure 5 shows that at day 150 only very few larvae had survived for the recording of the adult EOD. The same three specimens, measured at day 50, produced at day 150 a biphasic EOD (Fig. 7), which represents a typical juvenile EOD found in both parent species (Kirschbaum et al. 2016; Nguyen et al. 2017). The differences in the shape and the duration of the individual phases (Table 3) between the three individuals are very small. Due to the subsequent death of all three specimens, we were not able to follow the further ontogenetic development of the adult EOD of these specimens nor of additional specimens. The biphasic EOD comprising a first head positive and a second head negative phase indicates that the main stalk is located at the posterior face of the electrocyte (Bass 1986) (see also below).

As mentioned above, we were able to investigate histologically the anatomy of the adult electric organ in three specimens only due to the limited availability of the larvae due to death. Figure 8a shows a cross section of the adult electric organ of a 26.5 mm larva comprising and a single large stalk, surrounded by nerve tissue, extending into many smaller stalks. This is a typical feature of both parent species, *C. compressirostris* and *C. tamandua*, respectively (Paul et al. 2015).

The two 150 and 165 day old specimens analysed based on the sagittal sections (Fig. 8b–d) show a divergent EO structure. In one specimen (150 days old) there are penetrations as found in the F1-hybrids (Kirschbaum et al. 2016), whereas such penetrations are missing in the 165 day old specimen, which is typical for *C. compressirostris* (Paul et al. 2015). This could indicate a different allelic combination at underlying genes in these F2-specimens. However, sample sizes are still far too small for any conclusive inference regarding co-segregating allelic combinations and phenotypic traits. Once the general feasibility of F2-production has been demonstrated here, we will aim for such an analysis in future studies.

### Morphology of older larvae

The morphology of the three specimens whose EODs were investigated at day 150 was documented at the same time (Fig. 7). The pigmentation of the unpaired fins and of the body is different in all three specimens as is the morphology of the head. We suppose that these differences are due to new allelic combinations of the underlying genes in the F2-generation, as the pure species are characterized by a quite uniform morphology (Nguyen et al. 2017). Again, sample sizes were too small here to infer genes underlying these traits, which will be a definite goal of future studies.

## Conclusion

Our results show for the first time that F1-intragenus hybrids in mormyrids (*C. tamandua ♂ *× *C. compressirostris* ♀) are fertile and can produce F2-offspring and behave during reproduction like a valid species. However, the F2-hybrids did not survive for more than 5 months. The genetic distance between the two parent species is quite large (Feulner et al. 2007; Lamanna et al. 2016). We also obtained F1-hybrids between additional *Campylomormyrus* species (Kirschbaum et al. 2016), which are genetically more closely related to each other (Feulner et al. 2007; Lamanna et al. 2016). These F1-hybrids would be ideal candidates for further breeding experiments to produce more viable F2-fish and to enable co-segregation studies between phenotypic traits and their underlying genes. Such analysis is greatly facilitated by available genomic resources of several species, namely full transcriptomes (Lamanna et al. 2014, 2015) which have been systematically surveyed for species-specific expressed single nucleotide polymorphisms related to the EOD (Canitz et al. 2019).
