# Correction to: Intragenus F1-hybrids of African weakly electric fish (Mormyridae: *Campylomormyrus tamandua ♂ *× *C. compressirostris* ♀) are fertile

**DOI:** 10.1007/s00359-021-01513-2

**Published:** 2021-10-17

**Authors:** Yevheniia Korniienko, Linh Nguyen, Stephanie Baumgartner, Marianne Vater, Ralph Tiedemann, Frank Kirschbaum

**Affiliations:** 1 Faculty of Life Sciences, Albrecht Daniel Thaer-Institute of Agricultural and Horticultural Sciences, Unit of Biology and Ecology of Fishes, Humboldt University of Berlin, Philippstr. 13, Haus 16, 10115 Berlin, Germany; 2 Institute of Biochemistry and Biology, Unit of Evolutionary Biology/Systematic Zoology, University of Potsdam, Karl-Liebknecht-Str. 24-25, Haus 26, 14476 Potsdam, Germany; 3 Institute of Biochemistry and Biology, Unit of General Zoology, University of Potsdam, Karl-Liebknecht-Str. 24-25, Haus 26, 14476 Potsdam, Germany

## Correction to: Journal of Comparative Physiology A (2020) 206:571–585 10.1007/s00359-020-01425-7

The article “Intragenus F1‑hybrids of African weakly electric fish (Mormyridae: Campylomormyrus tamandua ♂ × C. compressirostris ♀) are fertile”, written by Yevheniia Korniienko, Linh Nguyen, Stephanie Baumgartner, Marianne Vater, Ralph Tiedemann and Frank Kirschbaum, was originally published Online First without Open Access. After publication in volume 206, issue 4, page 571–585 the author decided to opt for Open Choice and to make the article an Open Access publication. Therefore, the copyright of the article has been changed to © The Author(s) 2021 and the article is forthwith distributed under the terms of the Creative Commons Attribution 4.0 International License, which permits use, sharing, adaptation, distribution and reproduction in any medium or format, as long as you give appropriate credit to the original author(s) and the source, provide a link to the Creative Commons licence, and indicate if changes were made. The images or other third party material in this article are included in the article’s Creative Commons licence, unless indicated otherwise in a credit line to the material. If material is not included in the article’s Creative Commons licence and your intended use is not permitted by statutory regulation or exceeds the permitted use, you will need to obtain permission directly from the copyright holder. To view a copy of this licence, visit http://creativecommons.org/licenses/by/4.0. Open Access funding enabled and organized by Projekt DEAL.

The original article has been corrected.

